# Cost effectiveness of a novel device for improving resuscitation of apneic newborns

**DOI:** 10.1186/s12887-020-1925-5

**Published:** 2020-01-30

**Authors:** Ayman Ali, Jacob Nudel, Curtis R. Heberle, Data Santorino, Kristian R. Olson, Chin Hur

**Affiliations:** 1 Institute for Technology Assessment, Massachusetts General Hospital, Boston, USA; 2 Gastrointestinal Unit, Massachusetts General Hospital, Boston, USA; 3 Tulane University School of Medicine, New Orleans, USA; 4 Department of General Surgery, Boston University, Boston, USA; 5 Institute for Health System Innovation and Policy, Boston University, Boston, USA; 6 Mbarara University of Science and Technology, Mbarara, Uganda; 7 Consortium for Affordable Medical Technologies, Mbarara, Uganda; 8 Consortium for Affordable Medical Technologies, Massachusetts General Hospital, Center for Global Health, Boston, USA; 9 Harvard Medical School, Boston, USA; 10 Health Innovations Research and Evaluation (HIRE), Columbia University Medical Center, 630 W 168th Street, PH9 105, New York, NY 10032 USA

**Keywords:** Disability-adjusted life years, Mathematical model, Neonatal encephalopathy, Intrapartum-related hypoxia

## Abstract

**Background:**

Intrapartum-related hypoxic events are a major cause of morbidity and mortality in low resource countries. Neonates who receive proper resuscitation may go on to live otherwise healthy lives. However, even when a birth attendant is present, these babies frequently receive suboptimal ventilation with poor outcomes. The Augmented Infant Resuscitator (AIR) is a low-cost, reusable device designed to provide birth attendants real-time objective feedback on measures of ventilation quality during resuscitations and is intended for use in training and at the point of care. The goal of our study was to determine the impact and cost-effectiveness of AIR deployment in conjunction with existing resuscitation training programs in low resource settings.

**Methods:**

We developed a simulation model of the natural history of intrapartum-related neonatal hypoxia and resuscitation deriving parameters from published literature and model calibration. Simulations estimated the number of disability-adjusted life years (DALYs) averted with use of the AIR by birth attendants if deployed at the point of care. Potential decreases in neonatal mortality and long-term subsequent morbidity from disability were modeled over a lifetime horizon. The primary outcome for the analysis was the cost per DALY averted. Model parameters were specific to the Mbeya region of Tanzania.

**Results:**

Implementation of the AIR strategy resulted in an additional cost of $24.44 (4.80, 73.62) per DALY averted on top of the cost of existing, validated resuscitation programs. Per hospital, this adds an extra $656 to initial training costs and averts approximately 26.84 years of disability in the cohort of children born in the first year, when projected over a lifetime. The findings were robust to sensitivity analyses. Total roll-out costs for AIR are estimated at $422,688 for the Mbeya region, averting approximately 9018 DALYs on top of existing resuscitation programs, which are estimated to cost $202,240 without AIR.

**Conclusion:**

Our modeling analysis finds that use of the AIR device may be both an effective and cost-effective tool when used as a supplement to existing resuscitation training programs. Implementation of this strategy in multiple settings will provide data to improve our model parameters and potentially confirm our findings.

## Background

Intrapartum-related hypoxia (formerly referred to as birth asphyxia) is a major contributor to the global burden of morbidity and mortality with over 1 million cases of neonatal encephalopathy (NE) and over 700,000 deaths per year [1]. It drives nearly a half of neonatal mortality and is a major obstacle to achieving newborn health targets for the United Nation’s Sustainable Development Goal 3 and the specific goals set out in the Early Newborn Action Plan [2–5]. Globally, about 6% of newborns require basic resuscitation beyond stimulation and suctioning [1]. Hence, ensuring access to basic neonatal resuscitation is now a major priority in the delivery of global newborn health care [6].

**Fig. 1:**
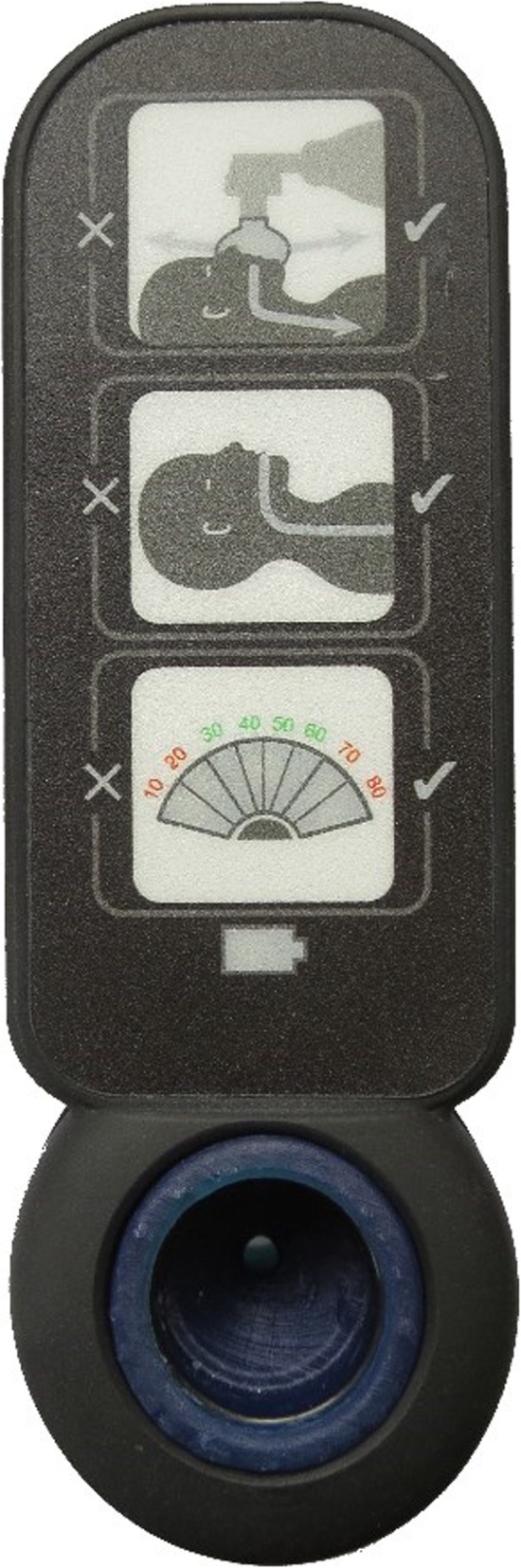
The Augmented Infant Resuscitator Device

The Augmented Infant Resuscitator (AIR) is a low-cost device that attaches in-line to widely available manual bag-valve-mask ventilation devices to monitor ventilation quality (Fig. 1). It can detect air leak, obstruction, hyperventilation, and hypoventilation. It has been systematically evaluated with precision equipment to very accurately determine these parameters on manikins and contribute only marginal additional air-path resistance [7]. The AIR also provides users real-time feedback through a color-coded LED display, potentially allowing birth attendants to improve the quality and outcomes of their resuscitative care. Additional details of the AIR are provided in the Additional file 1.

We developed and calibrated a mixed microsimulation-Markov model of apnea in newborns and used this model to assess the effectiveness and cost-effectiveness of deploying AIR at the point of care among birth attendants with the widely-deployed Helping Babies Breathe (HBB) training for improving the quality of bag-valve-mask resuscitation of non-breathing newborns in Sub-Saharan Africa [8]. Our primary endpoint was cost per disability-adjusted life year (DALY) averted, which is calculated relative to a control cohort receiving HBB training only.

## Methods

### Overview

To assess the implementation of AIR on long-term outcomes such as the morbidity and mortality that results from intrapartum-related neonatal hypoxia over a lifetime, a natural history model of the condition and subsequent sequelae was developed, calibrated, and analyzed. Model inputs and parameter estimates were derived from published literature or through calibration to reported outcomes. The model was structured as a mixed microsimulation-Markov model. Microsimulation was used to model adverse outcomes at birth and the potential for AIR to intervene upon the natural history of birth asphyxia; the Markov component was used to project initial outcomes across a lifetime to generate DALY estimates.

### Model structure and assumptions

**Fig. 2:**
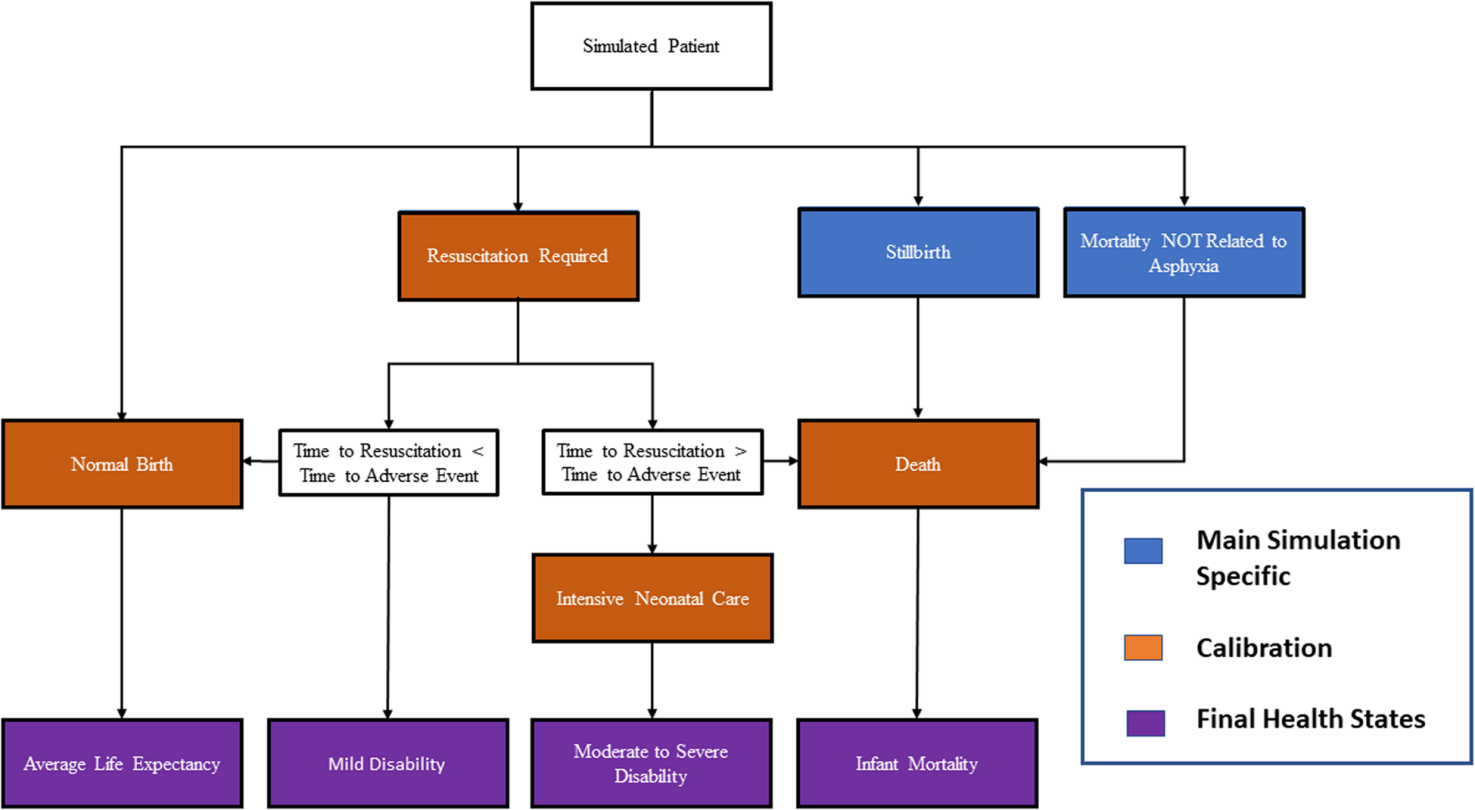
Model Schematic

We modeled the effect of deploying the AIR device at the point of care to assist with delivery of simulated cohorts of live-born babies born in sub-Saharan Africa. In our natural history, a microsimulation model, babies either breathe spontaneously, are stillborn, suffer a non-asphyxia related cause-of-mortality, or fail to initiate spontaneous respirations and require resuscitation (Fig. 2). For each simulated baby that required resuscitation, time-to-resuscitation and time-to-adverse-event values were drawn from separate, calibrated time-to-event probability distributions. When the time to an adverse event preceded the time to resuscitate, babies progressed from hypoxia to encephalopathy or death (via encephalopathy or cardiorespiratory collapse). When rescue preceded an adverse event, the baby was spared any further neurologic compromise secondary to cardiorespiratory failure.

In the event of a leak on the first mechanical ventilation, use of the AIR device was assumed to reduce the interval from initiation of bag valve mask (BVM) ventilation to recognition and correction of facemask leak by a fixed proportion. A 30% reduction was assumed given a recent randomized controlled trial with manikins has preliminarily shown a greater than 50% reduction in time to effective ventilation including absence of leaks, blockages, or incorrect rates [7, 9]. We performed a detailed sensitivity analysis on this variable, and we assumed that the device had no impact on any other time interval or model parameter during resuscitation attempts. In addition, we modeled the chance that a leak occurs after the initial correction. For such cases, we assumed the AIR had no net effect on outcomes. Further detailed description of the AIR device and our modeling of facemask leak is provided in the Additional file 1 (S1 Sections I - III). In our model, we only projected outcomes of the first year of implementation. The control cohort represented a facility in which HBB has been fully implemented. Therefore, our model results are presented specific to the AIR on top of existing HBB, which has been shown to have a highly favorable cost-effectiveness of 12–23 international dollars per DALY averted [10].

### Model inputs

Input parameters were estimated from published literature. Table 1 summarizes base-case values and the ranges used in sensitivity analyses. All costs are reported in USD (2014). In the Markov component of our analysis, there is a 3% annual discount rate applied [19]. Much of the clinical and cost data are from Tanzania, specifically the Mbeya region, and are assumed to broadly apply to Sub-Saharan Africa and potentially other low resource global settings. We included costs of implementing the Helping Babies Breathe program with and without the AIR device in Tanzania. These include administrative, training, and equipment costs estimated from a recent analysis by Chaudhury et al. that reported itemized costs of implementing HBB from a non-governmental organization perspective, summing to an average of $602.00 per facility [14].

**Table 1:** Model Input Parameters

Parameter Description	Value^a^	Source
Probability of a Leak	0.357	[11]
Probability of Leak after Initial Correction with AIR	0.222	[11]
Time of Leak (seconds)	Exponential (λ = 19.67)	[12]
Ventilation Rate per Minute	44.0	[13]
Probability of Apnea	0.0547 (0.01, 0.20)	[10]
Percent of Leak Time with AIR vs. Leak Time Control	70.0 (10.0, 90.0)	Model Assumption
Probability of Fresh Stillborn Births	14.4 per 1000 births	[10]
Cost of HBB Implementation (Control)	$602.00	[14]
Cost of an Additional Day of Training	$156.00	[14]
AIR Devices Required per Facility	4	[14] ^b^
AIR Device Cost	$125.00 ($100.00, $225.00)	^c^
Facility Traffic: Births per Year	4500 (3000–6000)	[10]
Life Expectancy Tanzania (Years)	64.944	[15]
Probability of Moderate to Severe Impairment Given Survived Asphyxia	0.269	[1]
Probability of Mild Impairment Given Survived Asphyxia	0.211	[1]
Disability Weight Moderate to Severe Impairment	0.42	[1, 16]
Disability Weight Mild Impairment	0.03	[1, 16]
Hazard Ratio Mortality of Moderate to Severe Impairment	11.36	[1, 17]
Hazard Ratio Mortality of Mild Impairment	3.15	[17]
Life Tables Tanzania	From 2012 Data	[18]

Key: SA (Sensitivity Analysis)

^a^Given as the base case value with a lower and upper bound tested in sensitivity analyses, if appropriate

^b^Based on the average number of providers trained per facility

^c^Manufacturer consultation

To project added costs of the AIR device training and usage, we estimated an additional required day of HBB training ($156) along with an added cost of four AIR devices, based on an average of four providers trained per hospital [14]. A cost of $125 per each AIR device was used based on pre-market estimates, which was our base-case value. In total, this added an additional $656.00 cost of implementation.

Disability weights assigned to patients with neurologic complications of NE were estimated from the published literature [1, 16]. Healthy babies were assumed to have life expectancy equal to that of the Tanzanian general population. Life expectancy among people with neurologic sequelae from NE was calculated from Tanzanian life tables using the mortality risk carried by patients with cerebral palsy and stratified by level of impairment, a technique used previously to estimate life expectancy among NE patients [1]. Calculation of DALYs was based on a simplified Markov model in which each state had only an annual transition probability to death.

### Calibration of natural history parameters

The relationships between intrapartum related hypoxic events, NE, and newborn death are incompletely understood and the transition probabilities between these health states are difficult to estimate directly from the published literature. Our assumption of time-to-event distributions for rescue and adverse events was the base of our natural history component. The time distributions were modeled with Weibull distributions and calibrated such that model output fit observed target data.

Our calibration component was restricted to the subset of patients for whom BVM is required and attempted (Fig. 2). We used the following calibration targets from the observational cohort study with the best available data characterizing the relationship between time-to-resuscitation and outcomes among babies requiring mechanical ventilation: (1) probability of asphyxia-related mortality; (2) mean time to resuscitation among survivors; (3) mean time to resuscitation among those who died; and (4) probability of initiation of BVM ventilation before 4 min [20].

For consistency, we used the same study that provided calibration targets to estimate the incidence and severity of NE in our simulated cohorts [20]. We assumed all of the non-breathing newborns in the study who survived but required hospital admission were moderately to severely disabled from NE. We calculated the number of apneic infants in the study who likely had mild NE using the relative incidence of mild-to-moderate versus severe NE in Sub-Saharan Africa [1]. Our assumption resulted in approximately 13/1000 simulated live births to be affected by NE. As verification, our modeled rates of NE match published estimates of the prevalence of NE in Sub-Saharan Africa [1]. Detailed methods utilized for optimization are provided in the Additional file 1 (S1 Sections II-IV). To address model uncertainty in the calibration, we acquired 50,000 unique sets of optimized parameters, sorted them by goodness of fit, and used the top 100 (0.2%) of sets in our final analysis. All calibrated parameter sets are provided in Additional file 1: Table S2.

Finally, after calibrating the underlying natural history, we included an additional calibration variable – probability of early, non-asphyxia related neonatal mortality (S1 Section IV). Neonates succumbing to non-asphyxia related mortality were assumed to derive no benefit from resuscitation attempts. Failure to properly simulate reported overall neonatal mortality in the region (HBB data, Additional file 1: Table S1) would result in an overestimation of the effectiveness of the AIR device and an underestimation of mortality in the region.

### Outcomes

The primary outcome of interest was the cost per DALY averted between cohorts of babies born to attendants with and without the AIR device. To determine the effects of model uncertainty, we performed extensive one-way sensitivity analysis on non-calibrated parameters. Because each analysis was run using the top 0.2% (100) of calibrated parameter sets, results are presented as an average value with a range. The range is a representation of the uncertainty in the model and is the minimum and maximum value model output from the various parameter sets. Our secondary outcome of interest was total cost of implementation in the Mbeya region, compared to the cost of HBB implementation alone. Mbeya is a region in southwest Tanzania with a 2012 population of 2.7 million.

## Results

A summary of the base case analysis and sensitivity analyses is provided in Table 2.

**Table 2:** Results of the Base Case and Sensitivity Analyses

Parameter Description (Base Case Value)	Direction	Value	Cost per DALY Averted (Min, Max)
Base Case (N/A)	N/A	N/A	24.44 (4.80, 73.62)
Proportion of Time of Leaking with AIR** (0.700)	Upper Estimate	0.85	51.27 (10.68, 510.02)
	Lower Estimate	0.05	7.75 (1.80, 16.59)
Hazard Ratio Mortality of Moderate to Severe Impairment (11.36)	Upper Estimate	20.00	23.39 (5.20, 61.75)
	Lower Estimate	2.50	26.43 (5.31, 109.50)
Hazard Ratio Mortality of Mild Impairment (3.15)	Upper Estimate	10.00	25.21 (4.66, 85.00)
	Lower Estimate	1.50	27.10 (4.59, 174.21)
Facility Births per Year	Upper Estimate	9000	11.76 (2.72, 36.81)
	Lower Estimate	2250	48.26 (10.83, 170.01)
Cost of AIR Device	Upper Estimate	250	44.63 (9.26, 129.06)
	Lower Estimate	100	20.43 (4.78, 72.92)
Probability of Apnea at Birth (0.0547)	Upper Estimate	0.109	11.68 (2.48, 28.51)
	Lower Estimate	0.027	52.48 (8.00, 259.71)

Key: DALY (Disability-Adjusted Life Year), AIR (Augmented Infant Resuscitator)

*Costs are presented as a mean value and a lower (minimum) and upper (maximum) bound that reflect the range of uncertainty in the calibrated parameter sets

**Defined as (t _AIR_ / t _NO AIR_), where t is the time spent leaking. Ratios closer to 0 are favorable for the AIR, as less time is spent leaking. Ratios closer to 1 reflect decreased effectiveness of the AIR, as the time spent leaking with the device is comparable to the time leaking without its use

### Base case analysis

In our base-case analysis, we found the AIR device implementation to result in average cost of $24.44 per DALY averted. Implementation of the AIR device is just slightly more expensive than the estimated cost of HBB itself, which has been estimated to cost about $12 to $23 per DALY averted [10]. When examined on a per-hospital basis, this amounts to approximately 26.84 years of disability averted in the assumed 3500 live births delivered at a cost of $656, on top of the $602 for HBB alone.

Chaudhury et al. calculate total roll-out cost of HBB of $202,240 for the Mbeya region in Tanzania, based on $602 per facility and 336 health facilities trained [14]. If AIR were simultaneously implemented, the total roll-out cost is estimated to be $422,688. However, using an estimate of 3500 live births per health facility, the added cost of $220,448 would be projected to avert approximately 9018 DALYs on top of the gain from HBB training alone. In addition, Chaudhury et al. project national roll-out of the program across Tanzania to cost $3,747,429 [14]; including the AIR in implementation would raise the cost to $7,832,308, but potentially may avert 167,106 DALYs.

### Sensitivity analysis

**Fig. 3:**
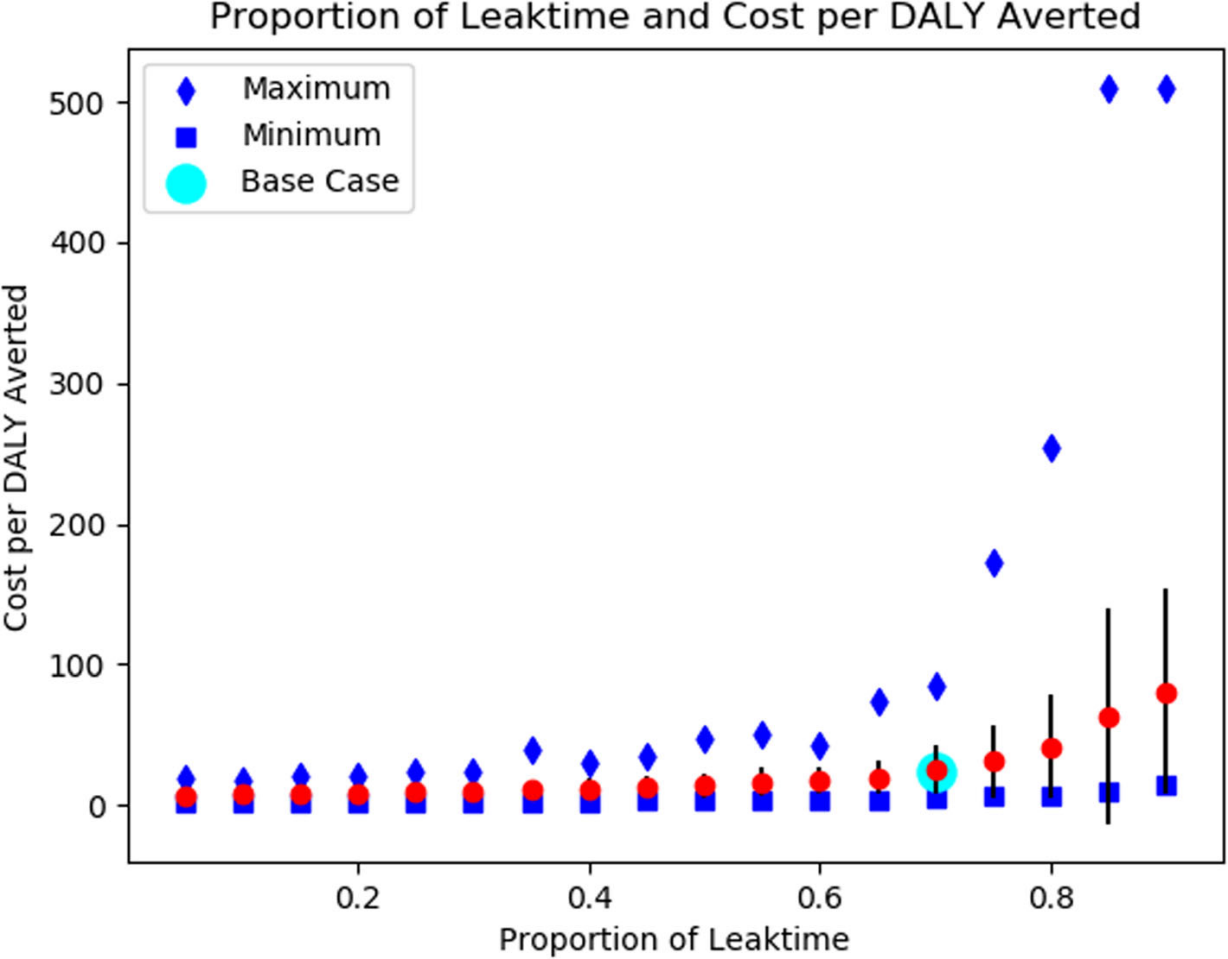
Probability of Apnea on the Impact of Augmented Infant Resuscitator Device

**Fig. 4:**
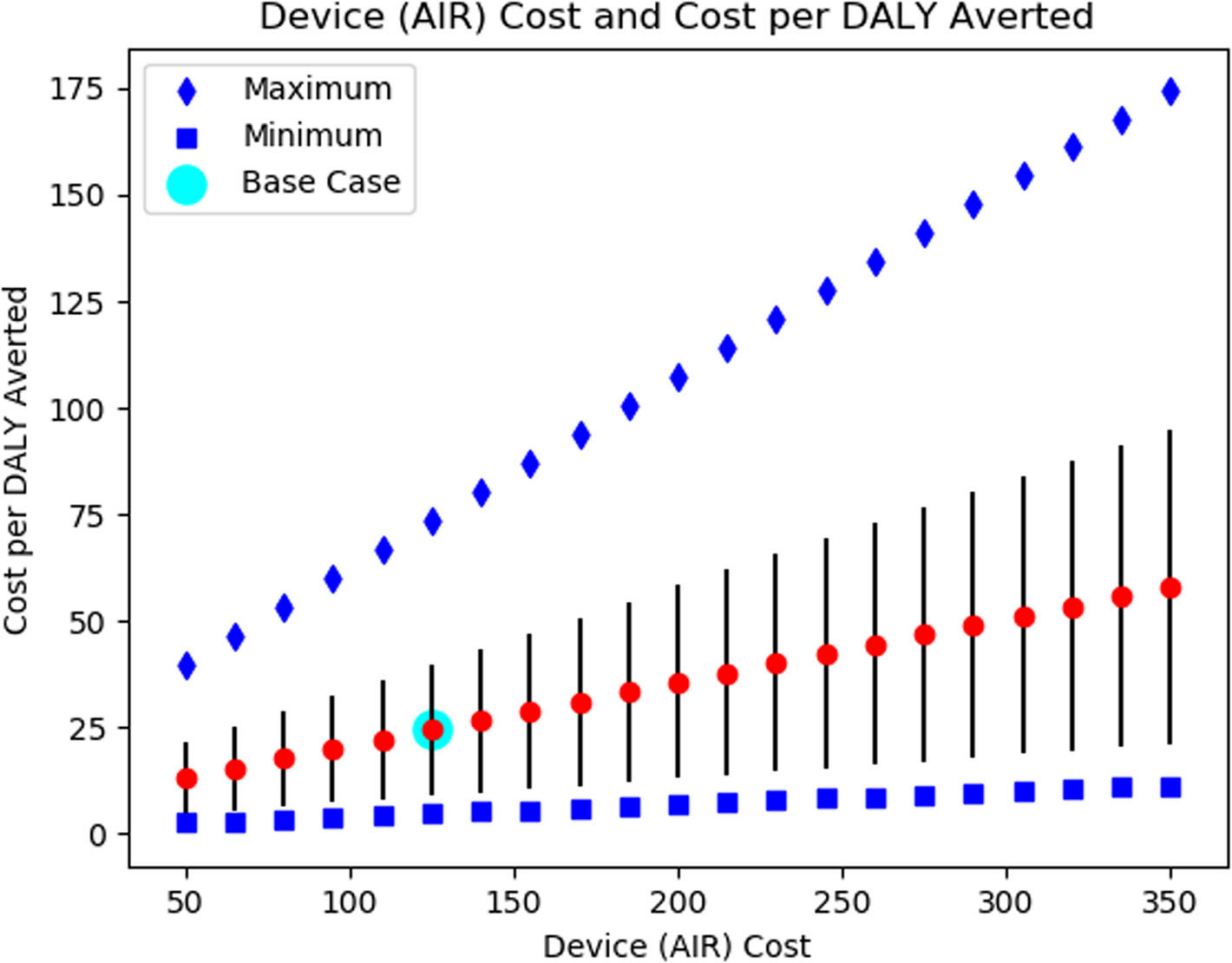
Reduction of Leak Time on the Impact of the Augmented Infant Resuscitator

**Fig. 5:**
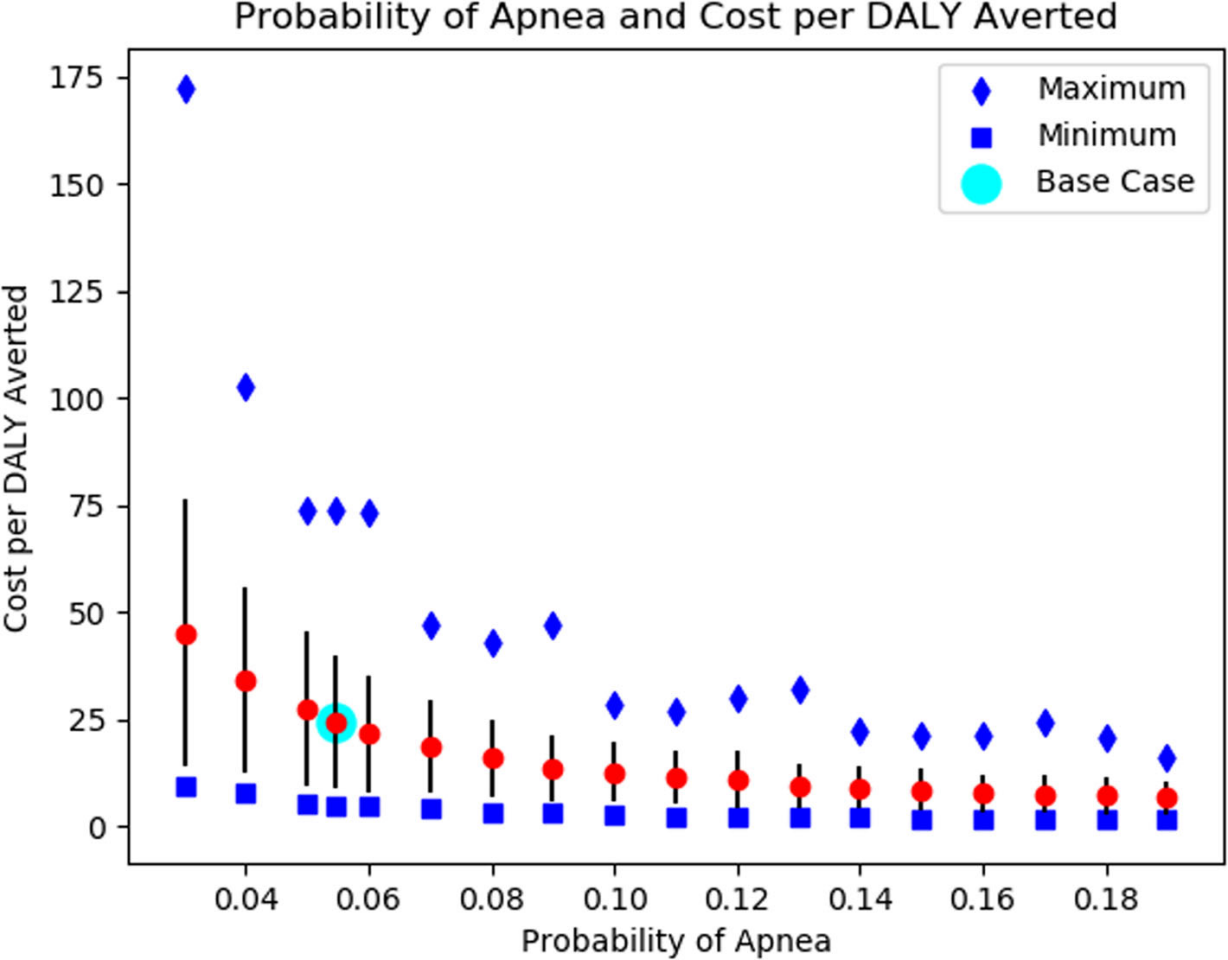
The Relationship of Cost of AIR and Cost per DALY Averted* Cost is done external to the model and does not require new random numbers, therefore the relationship depicted is linear

Our results were robust in all sensitivity analyses. In addition to upper and lower bounds on key parameters, we performed extensive analyses on the cost per DALY averted based on the probability of apnea, the percent reduction of the time spent leaking with use of the AIR, and the cost of the AIR device itself. The results of our analyses are shown in Figs. 3, 4 and 5.

## Discussion

Our model predicts a very cost-effective cost per DALY averted when using AIR in conjunction with well-established neonatal resuscitation training programs in Tanzania. In addition, it is estimated that it would cost $220,448 to implement the AIR across a large district in Tanzania to achieve approximately 9018 DALYs on top of the gain from HBB training alone. The cost effectiveness of a health intervention project is typically based on thresholds of 3X GDP per capita (cost-effective) or 1x GDP per capital (very cost-effective) [21, 22]. Tanzania has a GDP per capita of approximately $957.9. In 2017, Burundi had the lowest estimated GDP per capita of any country in the world at $286.0 – even in this setting, the AIR device remains well below the cost-effectiveness threshold. The total cost to bear from an NGO or governmental organization for AIR implementation with HBB across Tanzania would be $7,832,308, compared to $3,747,429 for HBB alone with 167,106 DALYs averted in the first year [14].

Recent physiologic evidence from Tanzania suggests that neonates with a diagnosis of either fresh stillbirth or early neonatal death in fact share a common pathophysiologic pathway from intrapartum related hypoxia to circulatory collapse. Neonates observed to be freshly stillborn are often misclassified as such [23]. Implementations of HBB in Tanzania and Indonesia have generally but not universally been associated with a reduction in the rates of both early neonatal death as well as fresh stillbirth, suggesting, perhaps, a shared hypoxic-ischemic pathway [2, 24–26]. As specific data is still limited, in our analysis we assume mortality due to fresh stillbirth and non-asphyxia related mortality are fixed. In other words, we include a mortality component that is not impacted with use of the AIR device.

Another strength of our study is our utilization of data from Haydom Lutheran Hospital (HLH), a site of rigorous data collection on individual births and resuscitation beginning prior to implementation of HBB training in 2009 and continuing through HBB implementation and improvement programs of the course over multiple years. By using statistics summarizing differences in resuscitation technique between babies who lived versus died at that site, we were able to model a plausible natural history explaining death and disability among non-breathing babies and offer a mechanism by which potentially improved resuscitation performance might improve outcomes among babies who sustain NE. We were also able to use observational data from human resuscitations to inform our estimate of the incidence and duration of facemask leak [11]. To our knowledge, this is the first attempt to mathematically model the natural history of intrapartum-related hypoxic events and their sequelae.

In one of Tanzanian study, researchers initially noted improved simulation performance among birth attendants after a one-day HBB training but no associated improvement in clinical outcomes [25]. Other studies have demonstrated HBB trainees experience rapid degradation of clinical skills acquired during training that may impede the transfer of skills acquired in training to clinical practice [8, 27, 28]. A statistically significant reduction in early neonatal mortality was, however, observed after implementation of “low-dose, high-frequency” training to improve skills and knowledge retention [25]. These results highlight the importance of continued, rigorous skills maintenance in neonatal resuscitation improvement programs. Though results are mixed, there is growing literature that technologies are supporting sustained behavior change in health care- for example, with real-time reminders boosting anti-retroviral adherence [29]. It is possible that the real-time feedback of the AIR device may help lead to durable practice change. However, this needs further investigation. In addition to the potential clinical gains that might be achieved through utilization of the AIR, the wireless data collection capabilities of the device can provide data on specific resuscitation performance parameters. Deploying the device in real resuscitations might thus elucidate relationships between resuscitation performance and clinical outcomes that have escaped current methods to date. This too will require investigation.

Our model focused on improving the quality of ventilation through accelerating the recognition of facemask leaks, which seem to be an important aspect of resuscitation quality in terms of delayed resuscitation. However, we did not account for the possibility that obstruction or incorrect rates of ventilation also causes ineffective resuscitation as suspected. Nor did we include the possibility that poor ventilation quality might lead to other outcomes, for example, barotrauma due to administration of inappropriately high-volume manual breaths. The AIR device is designed to indicate each of these parameters but were not included in this model which we believe may result in a conservative estimate of effect.

Like all modeling analyses, ours is limited by the data available to inform the model. Individual patient data was not available to inform calibration of survival curves among babies apneic at birth. Like Vossius et el 2014 [10], our reliance upon data from Haydom Lutheran Hospital, a single facility in rural Tanzania, may limit the generalizability of our findings to Sub-Saharan Africa. In addition, although we find that the AIR is likely cost-effective, implementation of the device would double the cost of HBB implementation. Furthermore, the AIR provides good but diminishing marginal returns over HBB alone. Further field testing is required to estimate the effectiveness of implementing the AIR device in programs with more limited training and experience as well as to determine if the AIR permits maintenance of skills to a degree that benefits outcomes greater over time. One-way sensitivity analyses demonstrated that results are most sensitive to variations in the cost of the AIR device. Thus, the cost of manufacture and delivery of the device must remain low to maintain cost-effectiveness as an increase in estimated cost by $100 results in an almost doubled cost per DALY averted. Also, ongoing consideration of the durability and maintenance of the device will be important to understand total costs and sustainability over time. Currently, the device design is for three years of normal, continuous use and to have a replacement rather than repair strategy.

## Conclusion

Our modeling analysis predicts a favorable cost per DALY averted ($24.44 [4.80, 73.62])) that is robust in sensitivity analyses. Total roll-out costs for AIR are estimated at $422,688 for the Mbeya region, compared to $202,240 for HBB without AIR, and we estimate averting approximately 9018 DALYs on top of the benefit of HBB. However, field tests of the AIR device are needed, and the estimated cost-effectiveness is less than that of HBB alone, suggesting prioritization of HBB implementation in birthing facilities. Clinical investigations should investigate the AIR device’s usability, durability, and safety at the point of care, potential to improve durability of neonatal resuscitation, and could confirm the benefits that our analysis found.

## Supplementary information

**Additional file 1:** Supplementary Information: Cost Effectiveness of a Novel Device for Improving Resuscitation of Apneic Newborns. **Table S1**. Calibration Targets. **Table S2**. Calibrated Parameters

## Data Availability

Not applicable.

## Abbreviations

AIR: Augmented Infant Resuscitator
BVM: Bag Valve Mask
DALYs: Disability-Adjusted Life Years
HBB: (Helping Babies Breathe)
HBB: Helping Babies Breathe
NE: Neonatal Encephalopathy
SA: Sensitivity Analysis
