# Supplementary material for: Cost effectiveness of a novel device for improving resuscitation of apneic newborns
Source: BMC Pediatr. 2020 Jan 30;20:46. doi: 10.1186/s12887-020-1925-5 (PMC6993372; doi:10.1186/s12887-020-1925-5)
Supplement: Supplementary file 1 — Additional file 1: Supplementary Information: Cost Effectiveness of a Novel Device for Improving Resuscitation of Apneic Newborns. Table S1. Calibration Targets. Table S2. Calibrated Parameters [file 12887_2020_1925_MOESM1_ESM.docx]

**This material is supplementary to:**

**Cost Effectiveness of a Novel Device for Improving Resuscitation of Apneic Newborns**

1. **The Augmented Infant Resuscitator (AIR)**

The AIR is a device that provides real-time feedback to users to improve newborn resuscitation. It attaches in line with most bag valve mask (BMV) and ventilation equipment. Potential use cases for the device include deployment at the point of care among birth attendants to help them improve resuscitation through faster recognition and correction of ventilation deficiencies; use in training to improve skill attainment and retention and to increase the likelihood of quality ventilation at the point of care; and quality improvement through data tracking to identify common problems with resuscitation training, equipment, and technique. An introduction to the user interface is provided below.


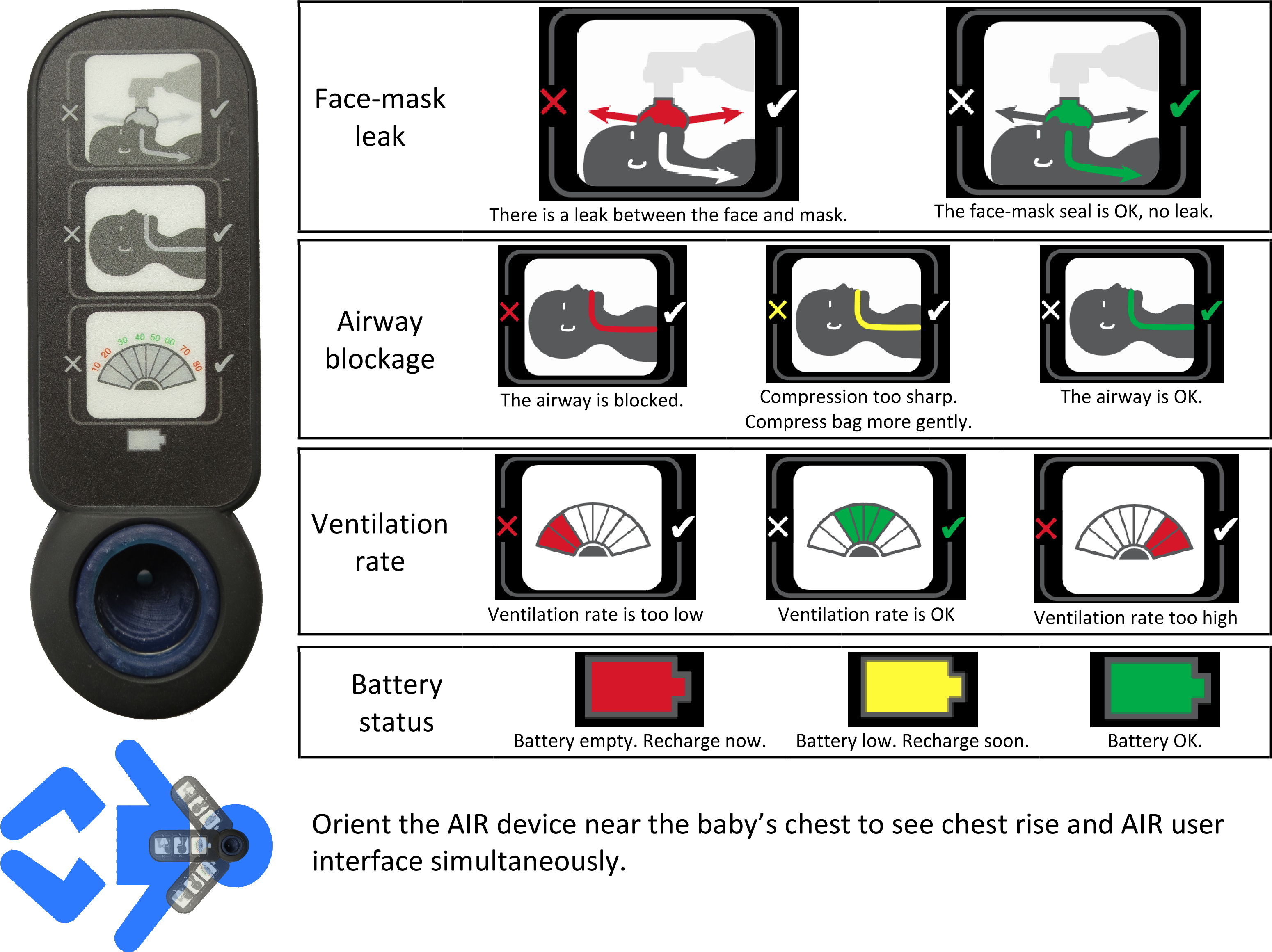


1. **Time to Event Distributions**

The time-to-adverse-event distribution for apneic newborns was modeled as a Weibull function. The time-to-rescue distribution was composed of the sum of three separate time-to-event probability functions modeled as follows: (1) a constant, parameter defining the delay between delivery and initial assessment;^a^ (2) a Weibull distribution representing an interval of assessment, diagnosis of apnea, suctioning and stimulation, and initiation of mechanical ventilation; (3) a binomial function representing the probability of facemask leak on the first ventilation; (4) in the event that there was a facemask leak on the first ventilation, an exponential function representing a delay between initiation of resuscitation and rescue during which time an operator recognizes and corrects facemask leak; in the event that there was no leak, this was assumed to be zero. Weibull distributions were employed in both above functions for their flexibility and theoretical ability to match calibration targets more closely than exponential distributions; for both distributions, we calibrated the shape and the scale parameters. They also allowed us to fit calibration parameters without imposing undue similarities in the shapes of time-to-event curves for adverse events and rescue. See table 1 for a summary of the calibration targets.

The probability of facemask leak on the first inflation and the number of ventilations prior to correcting the leak were estimated from an observational study of human resuscitations^1^ while the ventilation rate was estimated from a retrospective review of pediatric respiratory arrest simulations.^2^ The average duration of subsequent mask leak was estimated from these latter two values and used to model the probability of recognizing and correcting leak over time.

^a^ Author Assumption

1. **Optimization of the Natural History**

The natural history component of the model consists of the initial time-to-event simulation; i.e. time-to-adverse-event and time-to-rescue. Because the true distributions of the time-to-event functions were unknown, we approximated solutions by searching the parameter space with an optimization algorithm. The optimization iteratively minimized the chi-squared error between simulation outcomes and model targets. Each run of the optimization is continued until convergence on a parameter set.

We ran the optimization algorithm 50,000 times, thus obtaining 50,000 unique sets of parameters. The sets were then sorted by goodness of fit (chi-squared error) and, to address the uncertainty in the parameters, we ran the complete simulation using the top 0.2% (100) of the sets. This is similar to prior work using calibration that yields multiple good-fitting parameter sets.^3^ Each run of the optimization algorithm evaluates hundreds of simulations, each simulating 50,000 newborn requiring BMV. The ranges in our results represent the variability in the parameters of the natural history. Table 2 displays all 100 calibrated parameter sets used in the final analysis.

1. Calibration of Non-Asphyxia Related Mortality

Calibration with the methods described in sections II / III was based on outcomes of neonates with asphyxia at birth. Although this subset of patients accounts for a significant proportion of neonatal mortality, total neonatal mortality is significantly higher – estimated at 7.2 per 1000 live births.^5^ Therefore, it was necessary to include an additional parameter to include mortality unrelated to asphyxia. Without inclusion of non-asphyxia related mortality, our model would both underestimate mortality in the region, and overestimate the effectiveness of AIR and ventilation protocols (by assuming that all mortality was asphyxia related). The final calibrated value for non-asphyxia related mortality used in our model was 1.91 deaths / 1000 births.

**eTable I: Calibration Targets**

| **Parameter Description** | **Value** | **Source** |
| --- | --- | --- |
| Asphyxia Mortality | 9.8% | ^4^ |
| Time to initiation of BMV for Dead Patients | 100 seconds | ^4^ |
| Time to initiation of BMV for Survivors | 79.0 seconds | ^4^ |
| Percent Resuscitated before Four Minutes | 97.0% | ^4^ |
| Early Neonatal Mortality | 7.2 / 1000 | ^5^ |

**eTable 2: Calibrated Parameters**

| **Parameter Set** | **Time to Adverse Event, Weibull Distribution Parameters** | | | **Time to Resuscitation, Weibull Distribution Parameters** | | **Offset Time** |
| --- | --- | --- | --- | --- | --- | --- |
|  | **Shape** | **Scale** | **Shape** | | **Scale** |  |
| **1** | 1.00 | 613.85 | 0.85 | | 32.70 | 45.64 |
| **2** | 3.54 | 167.59 | 2.40 | | 61.75 | 27.13 |
| **3** | 1.01 | 606.06 | 1.56 | | 71.90 | 16.22 |
| **4** | 2.00 | 242.60 | 2.30 | | 78.36 | 11.22 |
| **5** | 3.84 | 161.97 | 2.63 | | 59.41 | 29.11 |
| **6** | 1.03 | 598.76 | 1.74 | | 77.16 | 12.81 |
| **7** | 1.98 | 254.69 | 2.83 | | 88.49 | 3.15 |
| **8** | 4.02 | 156.97 | 1.92 | | 47.18 | 38.22 |
| **9** | 2.57 | 203.99 | 2.69 | | 74.29 | 16.39 |
| **10** | 1.58 | 316.47 | 1.28 | | 44.60 | 40.79 |
| **11** | 2.30 | 220.82 | 2.01 | | 59.47 | 29.97 |
| **12** | 1.25 | 418.05 | 1.08 | | 42.49 | 39.36 |
| **13** | 1.02 | 592.22 | 1.15 | | 55.50 | 27.28 |
| **14** | 3.38 | 174.18 | 3.18 | | 87.44 | 4.01 |
| **15** | 3.07 | 178.39 | 1.53 | | 42.79 | 41.82 |
| **16** | 2.05 | 248.41 | 0.81 | | 22.13 | 56.19 |
| **17** | 1.99 | 248.63 | 2.35 | | 73.81 | 17.13 |
| **18** | 1.53 | 340.90 | 1.80 | | 63.62 | 25.18 |
| **19** | 3.43 | 172.95 | 2.69 | | 63.36 | 25.72 |
| **20** | 1.13 | 505.19 | 1.32 | | 62.42 | 22.10 |
| **21** | 1.40 | 369.72 | 2.12 | | 78.55 | 10.79 |
| **22** | 5.12 | 146.30 | 2.12 | | 42.45 | 46.19 |
| **23** | 2.46 | 207.90 | 1.47 | | 44.78 | 38.07 |
| **24** | 1.32 | 415.97 | 1.28 | | 51.55 | 33.21 |
| **25** | 2.50 | 212.55 | 3.63 | | 91.70 | 1.02 |
| **26** | 5.37 | 142.32 | 2.99 | | 70.42 | 16.01 |
| **27** | 1.18 | 499.74 | 2.37 | | 81.13 | 10.45 |
| **28** | 1.28 | 420.55 | 1.20 | | 47.09 | 37.73 |
| **29** | 6.70 | 131.11 | 2.49 | | 51.50 | 34.03 |
| **30** | 6.72 | 130.03 | 2.61 | | 55.77 | 30.21 |
| **31** | 2.22 | 219.46 | 1.43 | | 47.02 | 35.95 |
| **32** | 1.06 | 616.69 | 0.96 | | 38.51 | 42.45 |
| **33** | 1.87 | 261.56 | 1.96 | | 64.22 | 22.63 |
| **34** | 2.81 | 184.55 | 1.28 | | 35.60 | 45.36 |
| **35** | 0.99 | 689.70 | 1.66 | | 70.19 | 22.40 |
| **36** | 3.46 | 174.77 | 1.03 | | 22.88 | 62.03 |
| **37** | 1.51 | 345.06 | 2.18 | | 72.62 | 19.56 |
| **38** | 7.00 | 132.51 | 3.90 | | 72.29 | 18.19 |
| **39** | 1.64 | 299.77 | 2.62 | | 81.40 | 12.24 |
| **40** | 1.04 | 600.27 | 1.78 | | 73.57 | 18.96 |
| **41** | 3.23 | 174.24 | 2.76 | | 80.08 | 6.96 |
| **42** | 1.68 | 278.28 | 1.13 | | 41.25 | 39.20 |
| **43** | 2.62 | 205.35 | 3.95 | | 90.82 | 2.63 |
| **44** | 2.01 | 249.13 | 1.20 | | 37.14 | 44.22 |
| **45** | 1.64 | 307.84 | 2.04 | | 79.01 | 12.46 |
| **46** | 3.52 | 173.81 | 3.76 | | 89.99 | 4.57 |
| **47** | 1.79 | 289.73 | 2.97 | | 87.40 | 7.02 |
| **48** | 3.04 | 177.17 | 0.89 | | 18.69 | 58.67 |
| **49** | 3.30 | 183.86 | 3.10 | | 72.28 | 21.20 |
| **50** | 2.81 | 193.90 | 2.33 | | 74.01 | 12.98 |
| **51** | 1.79 | 275.78 | 2.41 | | 70.41 | 23.42 |
| **52** | 1.18 | 488.33 | 2.36 | | 86.46 | 8.99 |
| **53** | 7.00 | 129.83 | 4.57 | | 81.75 | 7.32 |
| **54** | 1.03 | 682.51 | 0.83 | | 26.39 | 55.76 |
| **55** | 2.95 | 195.11 | 1.98 | | 48.44 | 42.30 |
| **56** | 1.66 | 283.89 | 1.13 | | 39.75 | 39.00 |
| **57** | 6.78 | 127.12 | 1.68 | | 35.18 | 47.23 |
| **58** | 2.89 | 181.30 | 2.82 | | 84.11 | 1.92 |
| **59** | 1.00 | 573.59 | 0.82 | | 33.38 | 39.75 |
| **60** | 1.40 | 377.00 | 1.65 | | 53.91 | 37.52 |
| **61** | 1.28 | 436.71 | 1.62 | | 71.47 | 15.21 |
| **62** | 1.62 | 326.71 | 1.90 | | 60.89 | 32.32 |
| **63** | 1.08 | 594.46 | 1.49 | | 71.08 | 13.92 |
| **64** | 2.34 | 196.62 | 0.82 | | 19.15 | 59.56 |
| **65** | 1.49 | 329.56 | 1.81 | | 72.84 | 20.33 |
| **66** | 5.23 | 151.01 | 3.23 | | 65.39 | 27.89 |
| **67** | 5.92 | 146.03 | 3.57 | | 85.88 | 2.69 |
| **68** | 1.91 | 272.94 | 2.50 | | 71.12 | 22.30 |
| **69** | 2.03 | 249.08 | 2.34 | | 69.28 | 25.23 |
| **70** | 5.24 | 152.55 | 3.31 | | 81.14 | 8.47 |
| **71** | 1.11 | 513.54 | 1.21 | | 53.22 | 26.42 |
| **72** | 1.36 | 403.66 | 0.90 | | 25.19 | 59.32 |
| **73** | 2.60 | 204.19 | 1.12 | | 30.72 | 47.85 |
| **74** | 4.72 | 141.28 | 2.18 | | 50.61 | 32.62 |
| **75** | 2.51 | 213.83 | 3.17 | | 79.69 | 15.89 |
| **76** | 1.33 | 419.10 | 0.81 | | 29.06 | 48.74 |
| **77** | 6.29 | 141.55 | 1.57 | | 27.99 | 61.85 |
| **78** | 7.00 | 131.50 | 3.93 | | 60.70 | 30.50 |
| **79** | 2.58 | 185.25 | 1.22 | | 35.72 | 43.42 |
| **80** | 1.46 | 396.09 | 3.19 | | 86.22 | 7.52 |
| **81** | 1.61 | 291.17 | 2.06 | | 80.86 | 4.57 |
| **82** | 2.32 | 221.51 | 2.33 | | 51.31 | 39.97 |
| **83** | 2.05 | 230.09 | 1.25 | | 43.22 | 36.17 |
| **84** | 3.78 | 162.65 | 1.37 | | 25.88 | 63.17 |
| **85** | 1.01 | 642.24 | 0.90 | | 30.69 | 54.90 |
| **86** | 1.08 | 556.73 | 1.00 | | 32.11 | 54.95 |
| **87** | 1.36 | 391.58 | 3.06 | | 87.42 | 3.23 |
| **88** | 3.09 | 170.53 | 2.77 | | 83.34 | 4.21 |
| **89** | 4.71 | 142.67 | 2.41 | | 59.22 | 23.31 |
| **90** | 3.56 | 170.67 | 2.76 | | 74.82 | 9.75 |
| **91** | 1.57 | 358.62 | 0.70 | | 18.68 | 54.03 |
| **92** | 1.56 | 332.29 | 2.15 | | 71.83 | 14.46 |
| **93** | 1.08 | 599.75 | 2.78 | | 95.68 | 3.68 |
| **94** | 2.61 | 206.31 | 2.68 | | 59.53 | 34.78 |
| **95** | 1.32 | 435.79 | 2.76 | | 86.77 | 10.68 |
| **96** | 1.51 | 363.77 | 0.92 | | 20.24 | 65.29 |
| **97** | 1.07 | 587.79 | 0.62 | | 13.35 | 68.03 |
| **98** | 1.55 | 350.68 | 2.17 | | 84.30 | 12.55 |
| **99** | 1.31 | 405.20 | 2.84 | | 81.24 | 8.49 |
| **100** | 2.06 | 255.48 | 2.99 | | 90.33 | 7.17 |

References

1. Schmolzer GM, Dawson JA, Kamlin CO, et al. Airway obstruction and gas leak during mask ventilation of preterm infants in the delivery room. *Arch Dis Child Fetal Neonatal Ed* 2011;96(4):F254-7. doi: 10.1136/adc.2010.191171

2. Niebauer JM, White ML, Zinkan JL, et al. Hyperventilation in pediatric resuscitation: performance in simulated pediatric medical emergencies. *Pediatrics* 2011;128(5):e1195-200. doi: 10.1542/peds.2010-3696

3. Yeh JM, Kuntz KM, Ezzati M, et al. Development of an empirically calibrated model of gastric cancer in two high-risk countries. *Cancer Epidemiol Biomarkers Prev* 2008;17(5):1179-87. doi: 10.1158/1055-9965.EPI-07-2539

4. Ersdal HL, Mduma E, Svensen E, et al. Early initiation of basic resuscitation interventions including face mask ventilation may reduce birth asphyxia related mortality in low-income countries: a prospective descriptive observational study. *Resuscitation* 2012;83(7):869-73. doi: 10.1016/j.resuscitation.2011.12.011

5. Vossius C, Lotto E, Lyanga S, et al. Cost-effectiveness of the "helping babies breathe" program in a missionary hospital in rural Tanzania. *PLoS One* 2014;9(7):e102080. doi: 10.1371/journal.pone.0102080
